# Supplementary material for: Long Terminal Repeats of Gammaretroviruses Retain Stable Expression after Integration Retargeting
Source: Viruses. 2024 Sep 25;16(10):1518. doi: 10.3390/v16101518 (PMC11512309; doi:10.3390/v16101518)
Supplement: Supplementary file 1 [file viruses-16-01518-s001.zip › Supplementary_Materials.pdf]

Supplementary Information for

Long Terminal Repeats of Gammaretroviruses Retain Stable Expression After Integration Retargeting

SUPPLEMENTARY TABLES

SUPPLEMENTARY FIGURES

Supplementary Tables

Table S1. Pairwise comparison of median fluorescence intensity of transduced cells. The fluorescence intensity of K562 cells was measured at 3 dpi. Vectors expressed destabilized GFP.

	MoMLV			FeLV			SNV			KoRV			CrERV		
	wt	W390A	CBX	wt	W390A	CBX	wt	W390A	CBX	wt	W390A	CBX	wt	W390A	CBX
**MoMLV_wt**	1	1.1	1.2	1.8	1.6	1.2	1.2	1.4	1.8	5.7	5.6	9.4	9.1	9.9	9.6
**MoMLV_W390A**	1.1	1	1.1	1.6	1.4	1.1	1.1	1.3	1.6	5.3	5.1	8.7	8.4	9.1	8.8
**MoMLV_CBX**	1.2	1.1	1	1.5	1.3	1	1	1.2	1.5	4.7	4.6	7.8	7.5	8.2	7.9
**FeLV_wt**	1.8	1.6	1.5	1	1.1	1.4	1.5	1.2	1	3.2	3.1	5.3	5.1	5.6	5.4
**FeLV_W390A**	1.6	1.4	1.3	1.1	1	1.3	1.3	1.1	1.1	3.7	3.6	6.1	5.8	6.3	6.2
**FeLV_CBX**	1.2	1.1	1	1.4	1.3	1	1	1.2	1.4	4.7	4.5	7.7	7.4	8	7.8
**SNV_wt**	1.2	1.1	1	1.5	1.3	1	1	1.2	1.5	4.9	4.7	8	7.7	8.4	8.1
**SNV_W390A**	1.4	1.3	1.2	1.2	1.1	1.2	1.2	1	1.2	4	3.9	6.6	6.4	6.9	6.7
**SNV_CBX**	1.8	1.6	1.5	1	1.1	1.4	1.5	1.2	1	3.2	3.1	5.3	5.1	5.6	5.4
**KoRV_wt**	5.7	5.3	4.7	3.2	3.7	4.7	4.9	4	3.2	1	1	1.6	1.6	1.7	1.7
**KoRV_W390A**	5.6	5.1	4.6	3.1	3.6	4.5	4.7	3.9	3.1	1	1	1.7	1.6	1.8	1.7
**KoRV_CBX**	9.4	8.7	7.8	5.3	6.1	7.7	8	6.6	5.3	1.6	1.7	1	1	1	1
**CrERV_wt**	9.1	8.4	7.5	5.1	5.8	7.4	7.7	6.4	5.1	1.6	1.6	1	1	1.1	1.1
**CrERV_W390A**	9.9	9.1	8.2	5.6	6.3	8	8.4	6.9	5.6	1.7	1.8	1	1.1	1	1
**CrERV_CBX**	9.6	8.8	7.9	5.4	6.2	7.8	8.1	6.7	5.4	1.7	1.7	1	1.1	1	1

Table S2. Significance of differences in pairwise fluorescence intensity comparison. The fluorescence intensity of K562 cells was measured at 3 dpi. Vectors expressed destabilized GFP. First, the Kruskall-Wallace test was run. Then the significance of pairwise comparison differences was evaluated by Wilcoxon rank sum test with continuity correction. The statistical test was run in the R environment.

-log10 P-value	MoMLV		FeLV			SNV			KoRV			CrERV		
	W390A	CBX	wt	W390A	CBX	wt	W390A	CBX	wt	W390A	CBX	wt	W390A	CBX
**MoMLV_wt**	1.8	14.6	83.8	36.3	17.4	7.1	33.9	135.9	46.8	78.2	10.6	Inf	141.1	298.7
**MoMLV_W390A**	NA	9.2	82.5	31.5	11.9	3.7	28.5	156.5	45.9	78.2	10.6	Inf	146	Inf
**MoMLV_CBX**	NA	NA	48.4	12.9	0.4	0.3	10.5	96.8	41	71.9	10.1	Inf	141.2	Inf
**FeLV_wt**	NA	NA	NA	6.9	44	29.8	8.4	0	28	54.8	9.4	Inf	128.5	274.2
**FeLV_W390A**	NA	NA	NA	NA	10.5	9	0.2	10.4	32.6	60.8	9.7	Inf	129.4	268.9
**FeLV_CBX**	NA	NA	NA	NA	NA	0.7	8.8	93.8	38.7	68.1	9.8	Inf	139.2	Inf
**SNV_wt**	NA	NA	NA	NA	NA	NA	8	42.6	36.3	61.9	9.4	Inf	121	241.3
**SNV_W390A**	NA	NA	NA	NA	NA	NA	NA	12.6	32.5	59.3	9.5	Inf	126.4	260
**SNV_CBX**	NA	NA	NA	NA	NA	NA	NA	NA	29.1	58.1	9.4	Inf	138.6	Inf
**KoRV_wt**	NA	NA	NA	NA	NA	NA	NA	NA	NA	0.9	2.5	17.8	19.3	22.8
**KoRV_W390A**	NA	NA	NA	NA	NA	NA	NA	NA	NA	NA	2.3	18.7	20.6	25
**KoRV_CBX**	NA	NA	NA	NA	NA	NA	NA	NA	NA	NA	NA	0	0.5	0.3
**CrERV_wt**	NA	NA	NA	NA	NA	NA	NA	NA	NA	NA	NA	NA	4.2	4.6
**CrERV_W390A**	NA	NA	NA	NA	NA	NA	NA	NA	NA	NA	NA	NA	NA	0.6

Tables S3. Analysis of post-transduction expression intensities of AS.γRV.d2GFP vectors. (**A**) Fold change of median expression intensity of GFP+ cells. (**B**) Statistical significance of differences in expression intensities between vectors. First, the Kruskal-Wallis rank sum test was run giving a value of 77.9 ($-\log_{10}$ P-value). Then, the pairwise comparisons using the Wilcoxon rank sum test with continuity correction were run. The values represent the $-\log_{10}$ P-values.

A

Fold Change	MoMLV	SFFV	FeLV	SNV	CrERV	KoRV
**MoMLV**	1	1.1	1.2	2.2	2.7	5.1
**SFFV**	1.1	1	1	2	2.4	4.6
**FeLV**	1.2	1	1	1.9	2.3	4.5
**SNV**	2.2	2	1.9	1	1.2	2.3
**CrERV**	2.7	2.4	2.3	1.2	1	1.9
**KoRV**	5.1	4.6	4.5	2.3	1.9	1

B

-log10 P-value	CrERV	FeLV	KoRV	MoMLV	SFFV
**FeLV**	21.1	NA	NA	NA	NA
**KoRV**	13.4	35	NA	NA	NA
**MoMLV**	32	2.4	45	NA	NA
**SFFV**	16.8	0.4	28.7	1	NA
**SNV**	1.2	12.5	13.9	21.1	10.9

Supplementary Figures

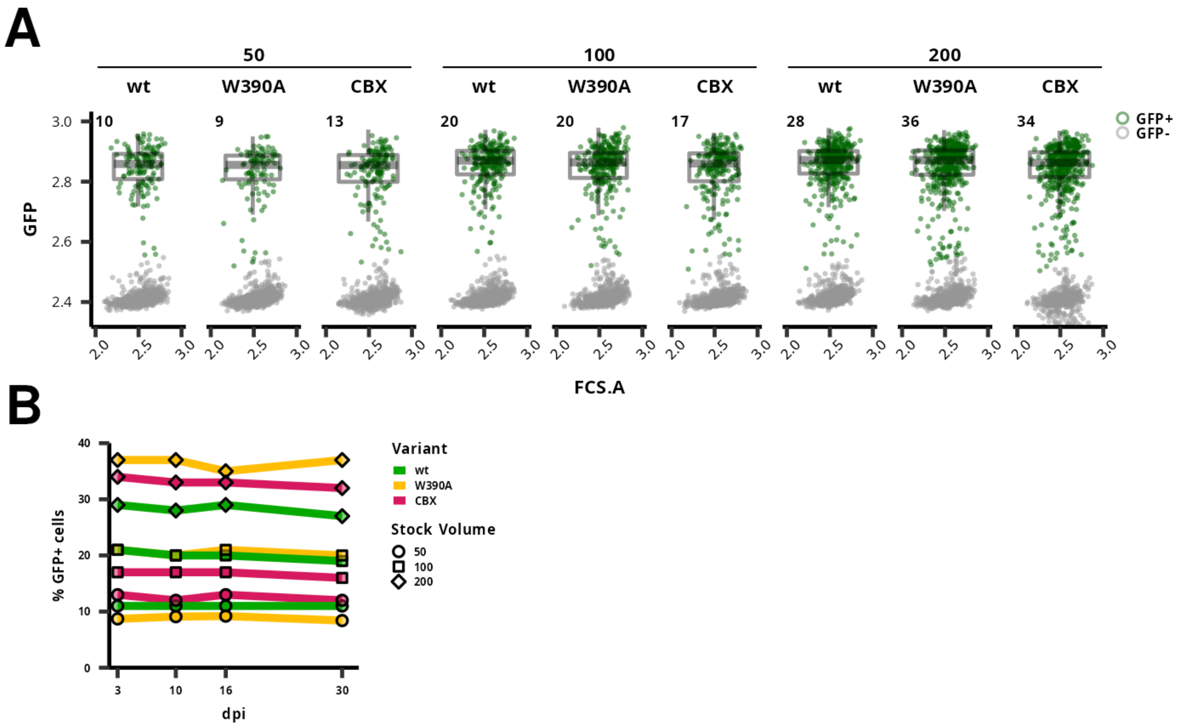

Figure S1. GFP expression intensity and stability by MLV-derived Bin vectors. (**A**) Intensity of GFP expression of MLV-derived vector with integrase variants at 3 dpi. K562 cells were transduced by a variable volume of LG vector viral stock and GFP expression was measured by flow cytometry at 3 dpi. The numbers above the graph represent the volume of the stock added. Smaller numbers mark the percentage of GFP+ cells (in green) in the transduced populations. Box plots show the distribution of GFP intensities among the GFP+ cells. (**B**) MLV-derived vector expression stability in time. The K562 cell line was transduced with 3 different volumes of the vector stock (in μL) represented by a point shape. Colors represent the IN variant of the vector used.

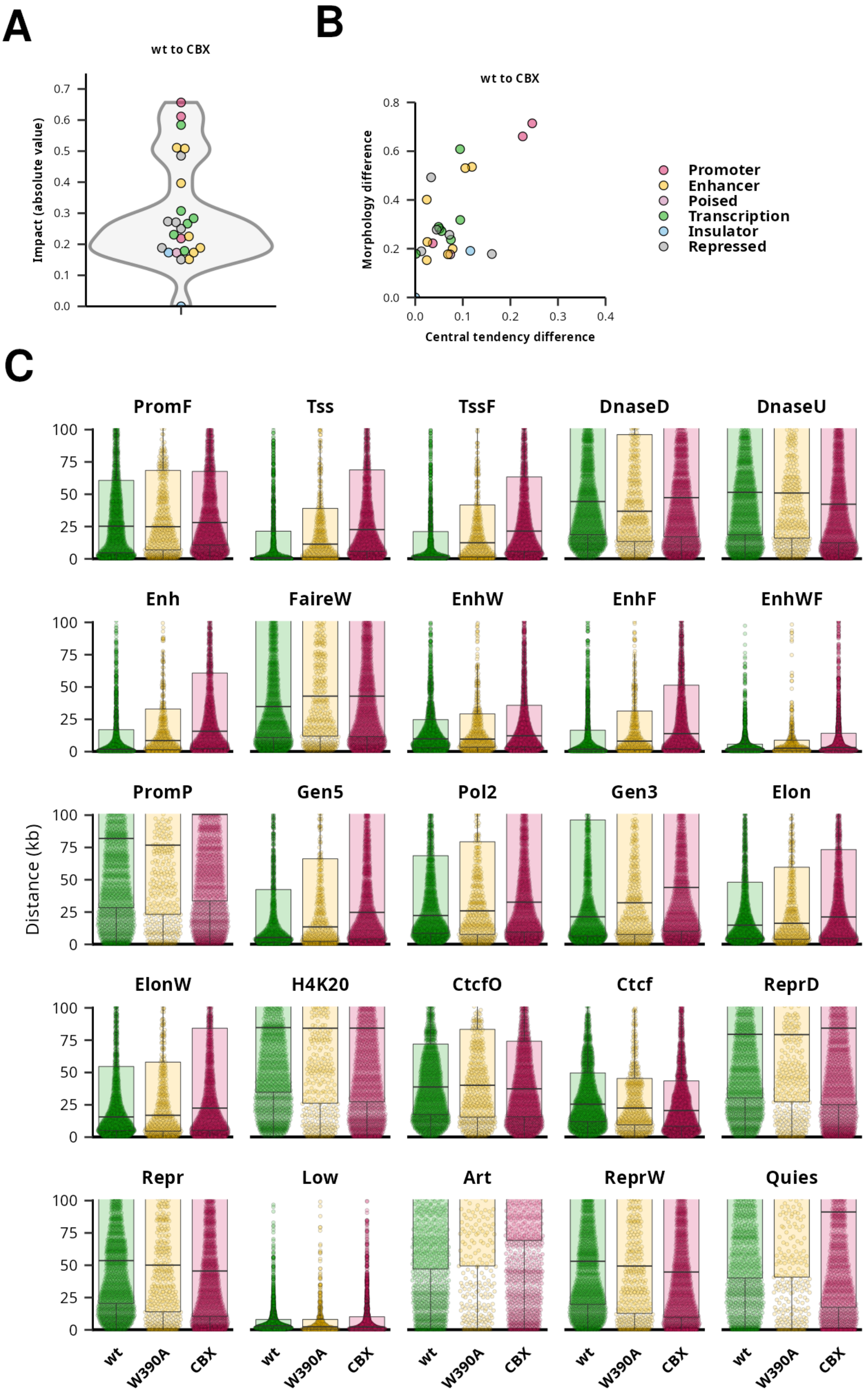

Figure S2. Distance of proviral IS to the nearest genome segment. (**A**) and (**B**) Effect size analysis between IS of IN^{wt} and Bin^{CBX}. Absolute values of Impact. B) Differences in central tendency and differences in morphology in distance distribution. C) Distances to the nearest genomic segment. Each IS is shown as a point.

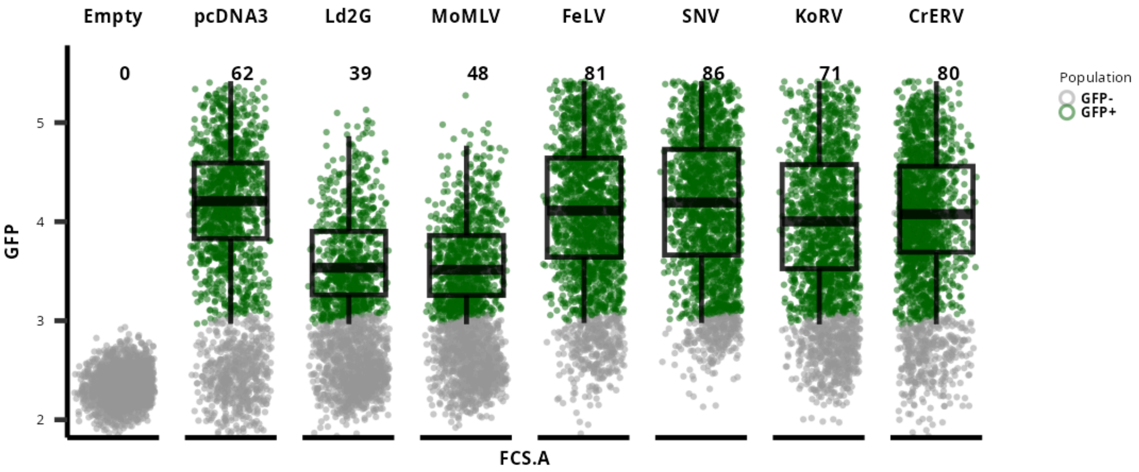

Figure S3. Expression of destabilized GFP (d2GFP) from gammaretroviral LTRs two days after transfection of HEK293T cells. Cells were transfected on a 24-well plate with 500 µg of the plasmid DNA using X-Treme Gene HP Transfection Reagent (Roche). 2,000 Hoechst-negative cells are shown for each sample. Boxplots mark the GFP intensity distribution of cells in the GFP-positive gate. The empty vector and pcDNA3 expressing d2GFP are used as positive and negative controls. pcDNA3 uses CMV as a promoter, Ld2G is a derivative from the LG vector. Names mark the origin of the LTR in LTR-d2GFP-LTR mini-vector used for transfection and subsequent vector production. The numbers in the upper part of the plot mark the percentage of GFP-positive cells from all Hoechst-negative cells.

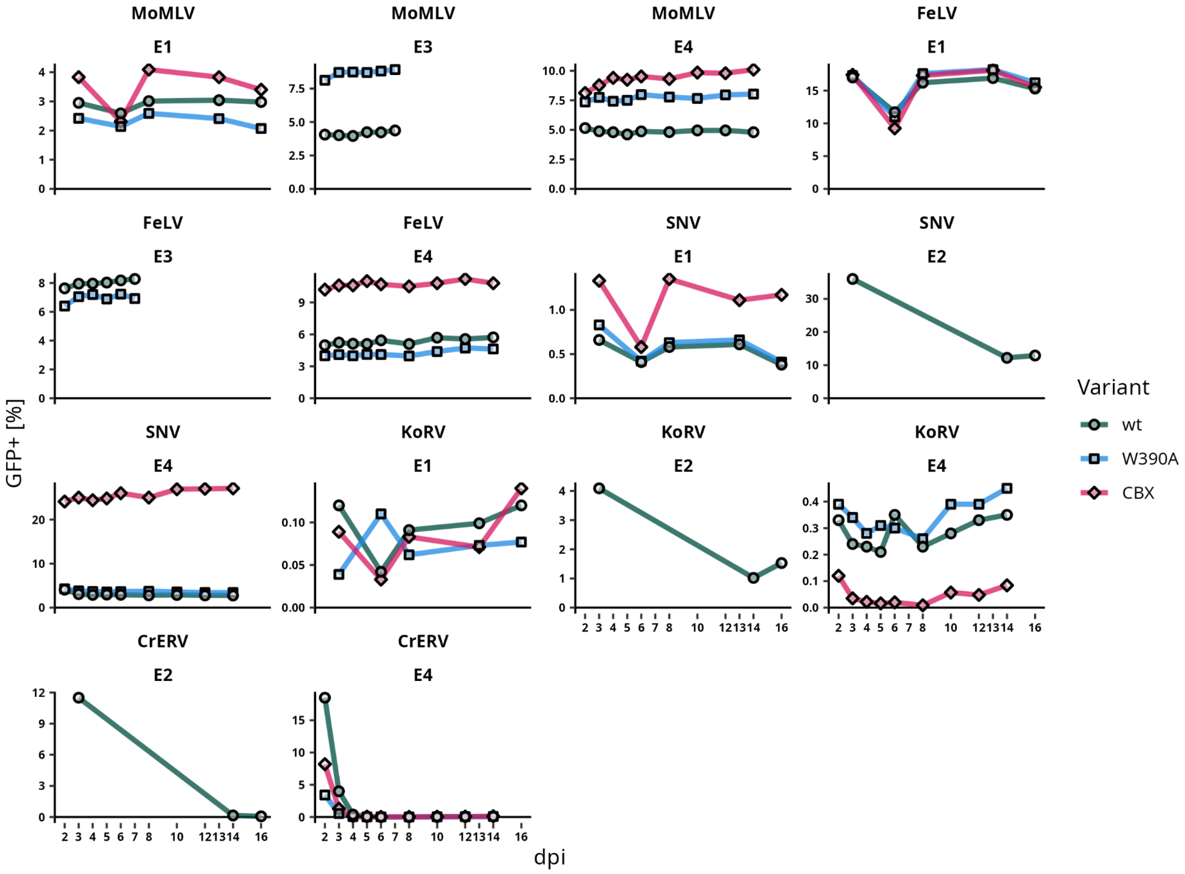

Figure S4. Gammaretroviral vector GFP expression stability in time. Each facet represents an individual transduction experiment performed with the gammaretroviral vector. The point shape and line color distinguish integrase variants.

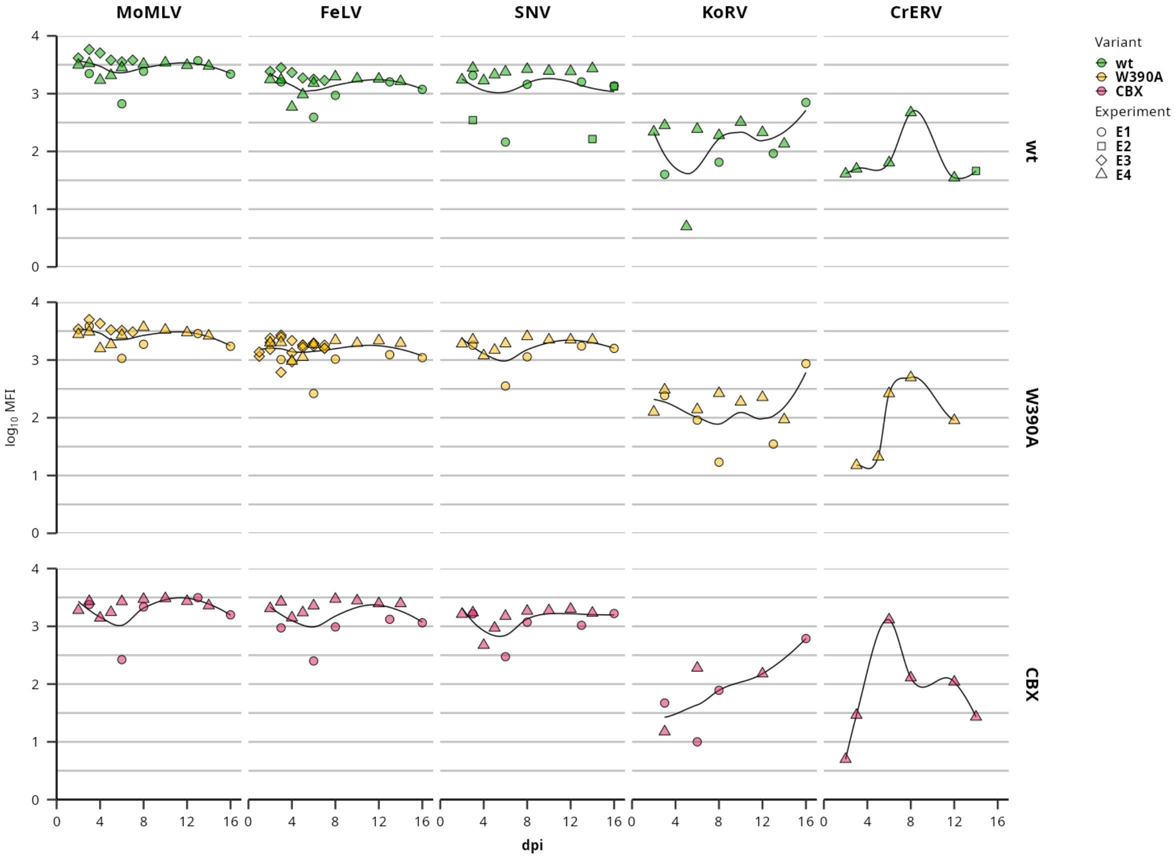

Figure S5. Gammaretroviral vector GFP expression intensity in time. Each column represents a gammaretroviral vector, and each row represents an integrase variant. Integrase variants are distinguished by the point color, the point shape indicates the transduction experiment. The smoothed line shows the intensity trend in time. Values of the GFP-positive population mean fluorescence intensities (MFI) are shown on the log_{10} scale.

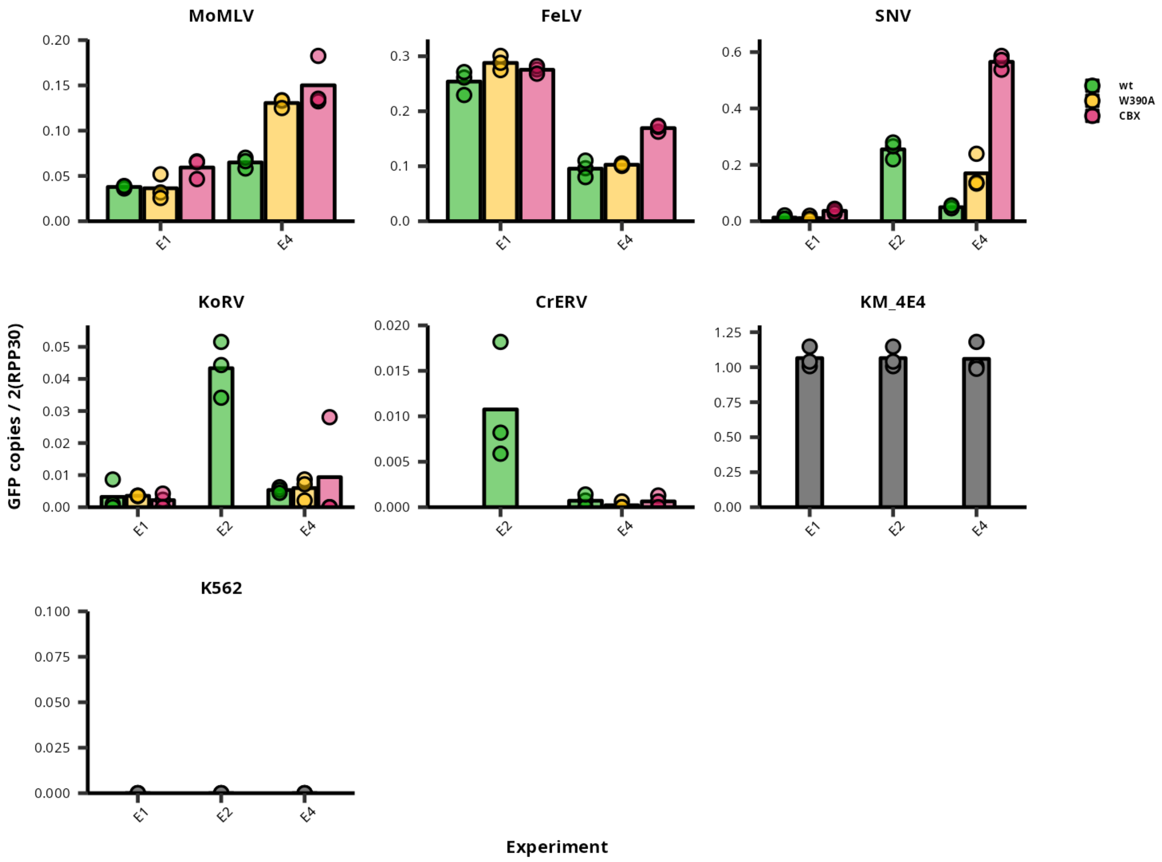

Figure S6. Copy number of gammaretroviral vector genomes. The copy number is a value obtained as a number of GFP copies divided by a doubled number of RPP30 copies - thus receiving a mean count of GFP copies per cell genomes. Each measurement was done in technical triplicate. KM_4E4 is a genomic DNA control obtained from a cellular clone transduced by MLV-derived GFP-expressing vector containing 1 copy of vector genome per cellular genome. K562 is a control cell population of the cell line not transduced by any vector.

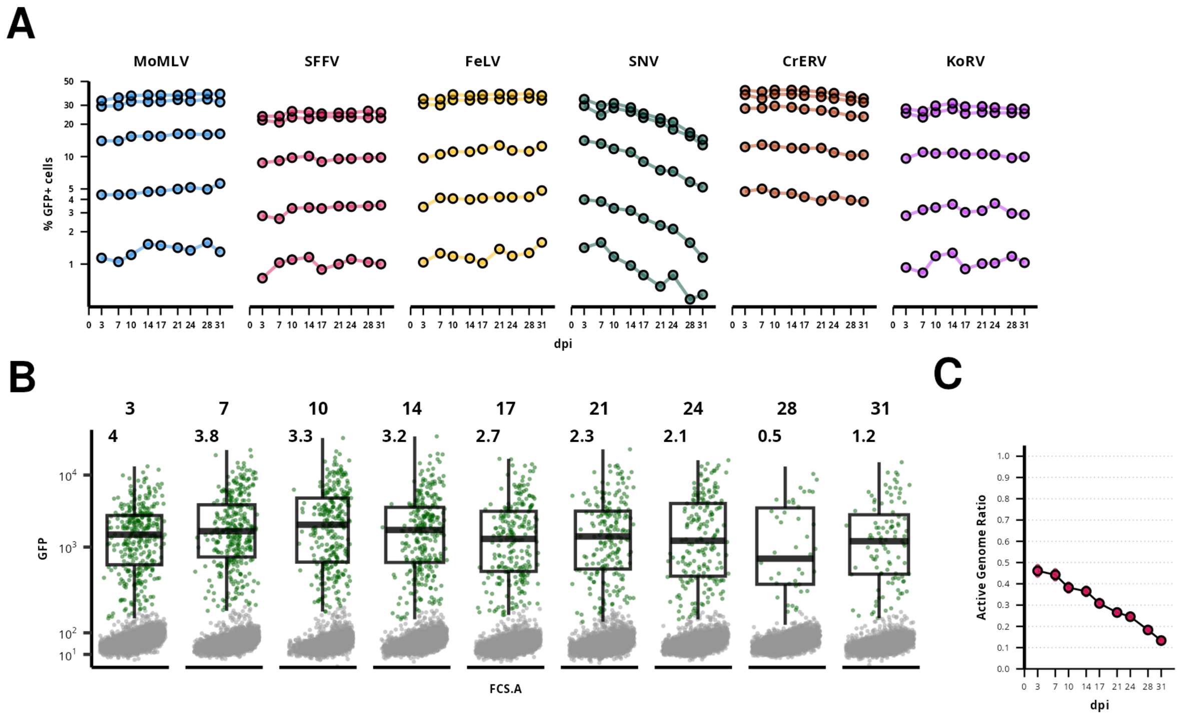

Figure S7. Gammaretroviral LTR activity as internal promoters in the alpharetroviral vector. (**A**) Percentage of GFP-positive cells in the K562 cell line. Each point represents a value obtained at a single measurement at a given dpi. Each line represents a single transduction experiment with various multiplicities of infection. GFP expression was followed from 3 dpi to 21 dpi. (**B**) Expression of the GFP by the vector with SNV LTR as an internal promoter. Upper numbers represent a dpi. Lower numbers represent a percentage of GFP-expressing cells. Boxplots represent the distribution of GFP intensity in a GFP-positive gate. (**C**) A ratio of GFP-positive cells and GFP copies per cellular genome. The GFP copy number was quantified on genomic DNA from cells collected at 14 dpi. The active genomes ratio was then obtained as a percentage of GFP-positive cells at a given dpi divided by a number of proviral copies per hundred cellular genomes at 14 dpi.
